# The Online Teaching Mode of College English in the Context of Gaussian Variant Genetic Algorithm

**DOI:** 10.1155/2021/9923364

**Published:** 2021-12-20

**Authors:** Shengfen Wang, Wei Hu, Yuan Lei

**Affiliations:** ^1^School of Foreign Language, Anhui Jianzhu University, Hefei, Anhui 230601, China; ^2^Economic & Management College, West Anhui University, Lu'an, Anhui 237012, China; ^3^School of Arts, Anhui Jianzhu University, Hefei, Anhui 230601, China

## Abstract

The current college English online teaching mode is mainly based on the traditional online MOOC teaching, which has some problems such as poor interaction. Under the mixed background, this paper studies the online college English teaching model based on the Gaussian mutation genetic algorithm and neural network algorithm. Firstly, it briefly introduces the general situation of network English teaching and the hybrid application of the Gaussian mutation genetic algorithm. Through the investigation and test analysis of students before and after class, the experiment evaluates students' network teaching quality in many aspects. On this basis, a better teaching quality evaluation model is proposed. Finally, the practical application shows that the model in this paper is very feasible. In the end, students have higher enthusiasm and seriousness in the hybrid context of college English online teaching based on the dual algorithm. English teaching quality can make use of each student's test scores in English classroom. This paper realizes the overall teaching through real-time dynamic tracking. Quantitative indicators are used to sort the influence degree of English classroom teaching indicators, which can effectively evaluate the quality of English classroom teaching.

## 1. Introduction

With the rapid development of science and technology in the 21st century, we have entered the global information age and then entered the education information age. The reform of education is closely connected with modern information technology. With the development and popularization of Internet technology all over the world, network education has become one of the most important innovations in the field of contemporary education. Because of its many advantages, network education gradually shows its strong vitality and great development potential. Therefore, network education has gradually been recognized by China's educational circles and has developed rapidly in the field of education. However, with the continuous advancement of network education, we also encounter more and more difficulties and problems to be solved. In order to make network teaching give full play to its own advantages and better serve education and teaching, several auxiliary means are used to supplement network teaching and promote the upgrading and transformation of the education industry.

Based on the Internet, network teaching can be carried out anytime and anywhere without the restriction of time and space. Learners can freely arrange the time, place, and progress of learning according to their actual situation, which enhances the convenience of learning and improves the flexibility of learning. At the same time, network teaching can popularize and share some advantageous education and teaching resources, and learners can easily obtain high-quality and massive teaching resources, which is conducive to the realization of national education and lifelong education.

At present, the mainstream teaching quality evaluation is still based on the traditional network teaching mode, while the online teaching mode is less [1, 2]. The emergence of a new generation of intelligent teaching methods, such as English online teaching mode and multidimensional data analysis teaching mode, provides rapid promotion of English online teaching in colleges and universities [3]. At present, online education of teaching quality has become an important content of modern evaluation [4]. Nowadays, there are many methods for teaching quality evaluation in colleges and universities. Students can conduct different online teaching according to different methods [5]. However, in today's many methods, it is difficult for students to carry out key training of knowledge points according to their actual situation and cannot achieve good learning effect [6].

The traditional online teaching mode of college English classroom can no longer meet the current objective requirements. Based on this, this paper studies the college English online teaching model in the mixed context based on the Gaussian mutation genetic algorithm and neural network optimization. According to the five factors affecting the quality evaluation of college English classroom teaching, this paper improves the traditional network teaching method. (1) The evaluation method proposed in this paper is more scientific. (2) Through practical tests, this paper quantitatively evaluates the reliability of the evaluation model of network teaching and English classroom teaching quality. The method proposed in this paper can not only effectively evaluate the effect of network teaching but also analyze and evaluate the current situation of college students' English ability. (3) The evaluation method of network teaching quality based on the Gaussian mutation genetic algorithm is proposed, which can effectively evaluate network teaching.

This paper studies the innovation of online college English classroom teaching mode and the construction of the quality evaluation system, which is mainly divided into four parts. The first section is a brief overview of the research background, research innovation, and chapter arrangement. The second section introduces the research status of online teaching mode and its influencing factors. In the third section, the online teaching quality evaluation based on the Gauss mutation genetic algorithm and neural network algorithm is constructed. According to Gauss random function and Laplace feature identification method, the online teaching quality evaluation model of the “genetic neural network” hybrid algorithm is built, and it is quantitatively characterized according to three dimensions of information [7]. The fourth section verifies the different indexes of the model proposed in this paper through relevant experiments.

## 2. Related Work

The development of the mode innovation is slow, while some countries have good foundation and stage innovation results in the field of online English teaching. Zhi et al. found that there are many problems in the design of different types of online English teaching modes [8]. Therefore, an intelligent online teaching strategy based on multiple objectives is proposed. According to participation of students in online English teaching mode, Entezami et al. put forward an online interactive teaching mode based on real-time pop-up strategy and neural network algorithm. The model has the characteristics of high interaction efficiency and strong stability [9]. Wang et al. combined with English practical teaching found that different types of students have obvious differences in the data diversity analysis in the process of learning English courses [10]. Therefore, a hierarchical ladder teaching mode is proposed. According to the rules of different spoken English training, Ruiz et al. put forward a better and targeted oral training method [11]. Ko et al. found that machine learning strategies can unify learning and management of English courses with similar contents [12]. Therefore, combining with the genetic algorithm, an online adaptive fusion online teaching mode of college English is designed. Delanoy and Kasztelnik conducted online and digital processing on different English listening materials and found that the efficiency of intelligent data information recognition of different types of listening materials is different and also affected by tone emotion [13]. According to the local optimization idea of the neural network algorithm, a grammar teaching system based on voice interactive training is proposed [14]. Zou et al. proposed a fractional online teaching algorithm, which can realize multimainline computing by combining the network association structure [15]. Haghrah et al. found that similar types of English articles have strong relevance in grammar. Therefore, an online English listening, speaking, reading, reading, and writing training method is proposed. This method can adaptively select the best training method according to the differences of English content, so it can effectively improve the students' English learning level [16]. Based on the existing online English teaching database, a new method of extracting the characteristics of English online teaching in mixed context is proposed based on the semantic differences of English phrases in different contexts. The results show that the method is more suitable for the context than the traditional method [17]. Li et al. made a differential analysis of the online English teaching system at present and unified the internal contact data according to the analysis results of data collection in the teaching process [18]. So et al. divided the online teaching process of college English into different data representation methods. Based on the optimization analysis of the Gauss mutation genetic algorithm [19], Liu et al. extracted the features of the existing English teaching methods and completed the internal relevance analysis and difference comparison based on the extraction results [17]. Based on the text information of the English test database in colleges and universities, Mikalef et al. made a differentiation analysis of the key English learning and proposed an online teaching strategy based on multiangle analysis. The experimental results show that the method can significantly improve the efficiency of students' review preparation [20]. Wilson found that there are great differences in online teaching mode of college English in different contexts. Therefore, an online teaching method based on adaptive changes of context is proposed [21]. To solve the problems of network Caton in the process of online English teaching, Kkese combined with 5 g data transmission technology and cloud storage technology proposed an end-to-end chaos method for online English teaching network, which effectively improved the video fluency and audio stability in the online English teaching process [22]. Cao et al. has developed a hybrid context online teaching method based on personalized recommendation according to different teaching characteristics and habits of English teachers, which can improve students' enthusiasm and participation in class to some extent [23].

In conclusion, we can see that the current teaching mode of English classroom in colleges and universities is mainly based on the differentiated teaching method, and it is rarely optimized by the intelligent algorithm and data analysis technology [24]. On the other hand, although great research results have been made in online English teaching, it has rarely been widely used, and few have good differential analysis model and evaluation model construction [25].

## 3. Methodology

### 3.1. Application of Gauss Mutation Genetic Algorithm to Optimize Neural Network Algorithm in English Online Teaching Model

In this study, the Gaussian algorithm is not only used to find the correlation between different data but can also explore and analyze the hot spots of data information. In addition, as a common algorithm, this algorithm is also the basic method of many data acquisition and analysis based on multi information. The Gauss mutation genetic algorithm is to achieve diversified feature matching and data group processing according to specific rules of target combination information and realize its internal uniqueness matching according to its internal relevance. In order to analyze the data types in different dimensions, its uniqueness needs to be distinguished according to the relevance of different data types and data groups. In order to realize the data analysis of the algorithm, the content of the target data is transformed into the pattern recognition content that can be analyzed by the computer. In this process, the vector space model is often used to process object feature data.

### 3.2. Implementation of Gauss Mutation Genetic Algorithm in College English Online Teaching Quality Evaluation Model

**Figure 1:**
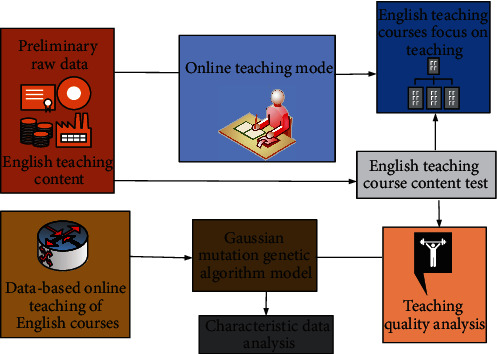
Online English teaching and quality analysis process.

This paper carries out a random survey on the learning process of college students in many schools, and the basic research objects are students of different majors. Through the survey on different aspects of most college students, finally, it analyzes and evaluates the results of different data on the evaluation of English teaching quality. This is also the basic implementation process of online teaching quality analysis for most college students. The process is shown in Figure 1.

The specific implementation process is as follows:

 
*Step 1*. Define a fitness function in the search space, given the population size, crossover rate, and mutation rate.
 
*Step 2*. Generate the initial population.
 
*Step 3*. Calculate fitness.
 
*Step 4*. If the termination conditions are met, exit; otherwise, go to step 5.
 
*Step 5*. Select Gaussian variation factor and copy multiple times. Generate subpopulation 1.
 
*Step 6*. Genetic factor crossover algorithm randomly determines multiple chromosomes and generates population 2.
 
*Step 7*. The genetic factor mutation algorithm generates population 3 according to the mutation number and then enters step 3 again.

**Figure 2:**
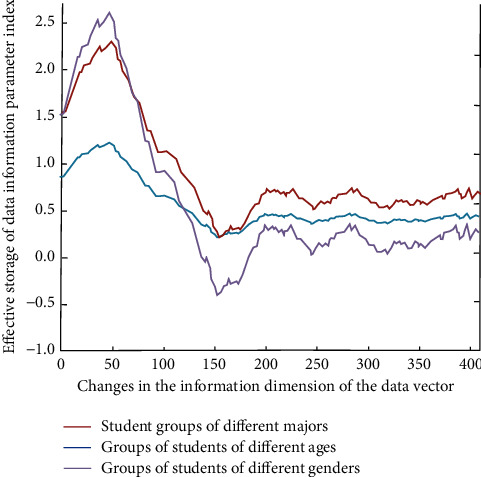
Effective storage of data information in different dimensions parameter indicators.

On the first hand, the first research data are the first survey data (mainly the record of the students' scores and punch cards in the English classroom teaching course) according to the current college English achievements of different types of students. In the online English teaching system, the data extraction of the listening status and online learning performance of each student is carried out. Then, the computer language processing is carried out by combining different types of multidimensional data information and converted into multichannel binary number so that the online teaching system can conduct quantitative analysis and feature extraction at the first time during multidimensional storage of students' learning data information. The results are shown in Figure 2.

With the increase of the dimension of the transformation from teaching information to data vector information, its change law is more obvious. The change law is more obvious, and almost all of them reach the minimum value at the middle and front position (150), which is also in line with the law of data analysis.

**Figure 3:**
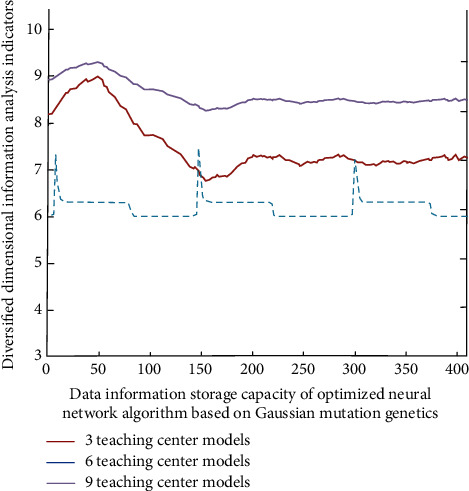
Data dimensional analysis simulation results under different online teaching center models.

Secondly, in this study, through the analysis of the characteristics of online listening students and online teaching English teachers, and according to their roles, some online questionnaires are carried out, such as the design of students' questions in English reading ability, writing ability, oral expression ability, and online teaching seriousness. The best online teaching scheme and learning strategy are extracted quickly, then the optimal scheme is processed by data, and its data information is imported into the online teaching system. Then, the variable dimension information is obtained according to its diversified coupling degree information, so that on the basis of the original, it is divided into different online teaching center models according to its characteristics. The online teaching simulation analysis results are shown in Figure 3.

As can be seen from Figure 3, the optimal online teaching scheme has different types of optimization degree improvement and shows a gradual increase and then gradually stable trend.

**Figure 4:**
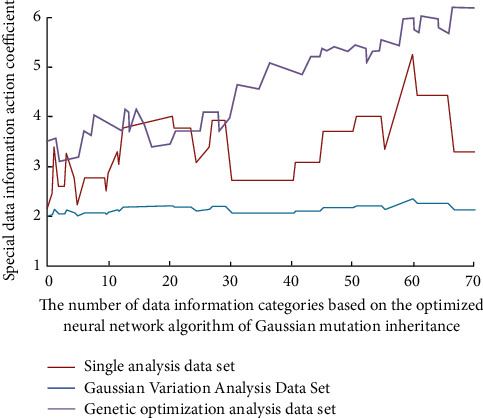
Special data information function coefficients under different data information classifications.

The other is to delete some unimportant information. The vector recording method is used to collect these meaningless data and form records. Therefore, these collected data can be converted and stored as vector information. When the coincidence degree meets the preset requirements, it can realize the data processing, judgment, and classification of the target data. The simulation results are shown in Figure 4.

As can be seen from Figure 4, with more data information classification, the more special data information inside will play a role, the internal differentiation will be more obvious, and the trend of its law will also have more obvious differences. In the process of highly similar calculation, the distance and angle between different vectors will have a certain relationship. The smaller the distance and angle, the higher the similarity of the information of the two algorithms. and the higher the positive correlation between English theoretical knowledge and the actual English level.

Let *x*_*i*_=(*x*_*i*1_, *x*_*i*2_,…, *x*_*ip*_) and *x*_*j*_=(*x*_*j*1_, *x*_*j*2_,…, *x*_*jp*_) be the online English learning observations of different students, then the similarity measure function *δ*(*x*_*ij*_) and relevance function *γ*(*x*_*ij*_) between them can be expressed as follows:

(1) $$\begin{array}{c} \delta \left(x_{ij}\right)=\frac{1}{\sqrt{\left[\sum_{k=1}^{p}{\left(x_{ik}-{\bar{x}}_{i}\right)}^{2}\right]\left[\sum_{k=1}^{p}{\left(x_{jk}-{\bar{x}}_{j}\right)}^{2}\right]}},\\ \gamma \left(x_{ij}\right)=\sum_{k=1}^{p}\left(x_{ik}-{\bar{x}}_{i}\right)\left(x_{jk}-{\bar{x}}_{j}\right). \end{array}$$

The angle cosine cos  *θ* corresponding to each angle is as follows:

(2) $$\begin{array}{c} \cos_{\delta }\theta =\frac{1}{\sqrt{\sum_{k=1}^{n}x_{ki}^{2}+\sum_{k=1}^{n}x_{kj}^{2}}},\\ \cos_{\gamma }\theta =\sum_{k=1}^{n}\frac{x_{ki}+x_{kj}}{2}. \end{array}$$

The corresponding Euclidean distances *d*_*ij*_ are as follows:

(3) $$\begin{array}{c} d_{ij}\delta =\sqrt{\frac{1+\left(\sum_{k=1}^{n}x_{ki}^{2}+\sum_{k=1}^{n}x_{kj}^{2}\right)}{n}},\\ d_{ij}\gamma =\sqrt{\frac{1+\left(\sum_{k=1}^{n}\left(x_{ki}+x_{kj}/2\right)\right)}{n}}. \end{array}$$

The sum of square difference *s*^2^ corresponding to each of them is as follows:

(4) $$\begin{array}{c} s_{\delta }^{2}=\frac{1}{n}{\sum_{k=1}^{n}\left[\sum_{k=1}^{n}x_{ki}^{2}+\sum_{k=1}^{n}x_{kj}^{2}\right]}^{2},\\ s_{\delta }^{2}=\frac{1}{n}{\sum_{k=1}^{n}\left[\sum_{k=1}^{n}\frac{x_{ki}+x_{kj}}{2}\right]}^{2}. \end{array}$$

### 3.3. Optimization Process of Gauss Mutation Genetic Algorithm and Neural Network Algorithm in Online English Teaching

The Gauss mutation genetic algorithm used in this paper has many advantages, such as in the speed and accuracy of data processing, it has good controllability and precision analysis, while in the diversified data categories, it maintains a low error rate and fault tolerance rate.

The optimized neural network algorithm of the Gaussian mutation genetic algorithm is composed of units in each layer, and its process includes the following parts:

 
*Point 1*. The input layer is the instance feature vector of the training set
 
*Point 2*. The weight of the connection point is passed to the next layer, and the output of one layer is the input of the next layer
 
*Point 3*. The number of hidden layers can be arbitrary, the input layer has one layer, and the output layer has one layer
 
*Point 4*. Each unit can also be called a nerve node, defined according to biological sources
 
*Point 5*. The above becomes a two-layer neural network, and the input layer is not counted in it
 
*Point 6*. Summation is weighted in one layer and then the output is transformed according to the nonlinear equation

**Figure 5:**
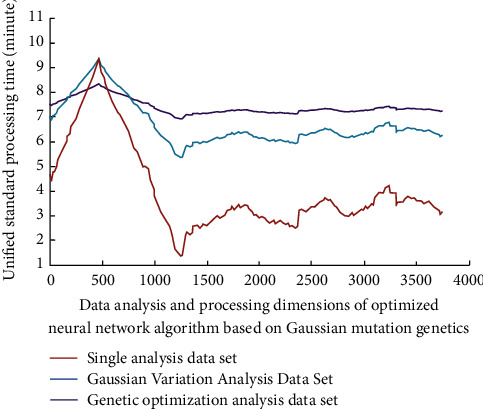
Unified standard processing time required under different data dimensions.

On the other hand, in the process of intelligent analysis in the mixed context of online English teaching, it needs to classify and process the students' English level, English application ability, and diversified intelligent data. In the process of classification, it needs to select stable strategies according to their differences and then combine with different multiple thinking modes. After several times of training and threshold determination, the stability data with different differences are processed online and standardized. The data analysis and processing process is shown in Figure 5.

In Figure 5, with the increase in the dimension of data analysis and processing, within a certain range (0–500), the unified standard processing time is also longer. This is because the larger the dimension is, the larger the data volume is, and the single processing data volume is effective. Therefore, the larger the dimension is, the more the processing time is. The Gauss mutation genetic algorithm can carry out differentiation analysis according to the content of English online teaching, and its internal relevance is expressed by the diversification analysis function, which is expressed as follows:

(5) $$\begin{array}{c} H\left(x\right)=\frac{9x^{7}+9x^{5}+5x^{3}+3x^{2}+2}{7x^{7}+3x^{5}+5x^{3}+1}, \end{array}$$

where *x* is the type of quantity group to be processed. The Gauss mutation genetic algorithm will carry out the uniqueness analysis according to its inherent data differences, while the analysis of data relevance is through the comprehensive discrimination of analytic function *μ*(*x*_*ij*_), coupling function *η*(*x*_*ij*_), and correlation function *α*(*x*_*ij*_), whose expressions are as follows:

(6) $$\begin{array}{c} \mu \left(x_{ij}\right)=\frac{\sum_{k=1}^{n}\left(\left(x_{ik}{\bar{x}}_{i}/i\right)+\left(x_{jk}{\bar{x}}_{j}/j\right)\right)}{{\bar{x}}_{i}+{\bar{x}}_{j}},\\ \eta \left(x_{ij}\right)=\frac{\sum_{k=1}^{n}\left(x_{ik}-{\bar{x}}_{i}\right)\left(x_{jk}-{\bar{x}}_{j}\right)}{i+j},\\ \alpha \left(x_{ij}\right)=\frac{1}{\sum_{k=1}^{n}\left(x_{ik}{\bar{x}}_{i}+x_{jk}{\bar{x}}_{j}/i+j\right)}. \end{array}$$

The threshold values of information degree *R* corresponding to the three functions are as follows:

(7) $$\begin{array}{c} R_{\mu }=\frac{\sum_{k=1}^{n}\left(\sqrt{\left(x_{ik}{\bar{x}}_{i}/i\right)+\left(x_{jk}{\bar{x}}_{j}/j\right)}\right)}{i^{2}+j^{2}},\\ R_{\eta }=\frac{\sum_{k=1}^{n}\sqrt{\left(x_{ik}-{\bar{x}}_{i}\right)\left(x_{jk}-{\bar{x}}_{j}\right)}}{i^{2}+j^{2}},\\ R_{\alpha }=\frac{i^{2}+j^{2}}{\sum_{k=1}^{n}\left(\sqrt{\left(x_{ik}{\bar{x}}_{i}+x_{jk}{\bar{x}}_{j}/i+j\right)}\right)}. \end{array}$$

**Figure 6:**
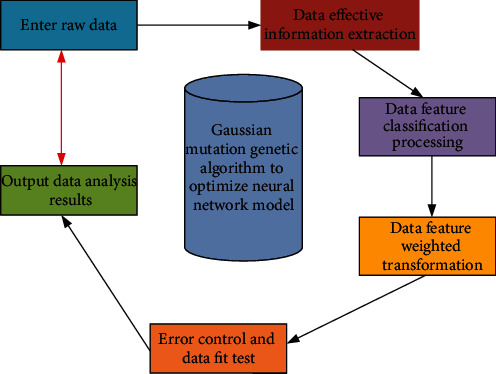
Gaussian mutation genetic algorithm optimizes the data processing simulation process of the neural network model.

Among them, *x* is the type of quantity group to be processed, *n* is the total number of weighted data, and *k* is the total number of weighted current data. After the above analysis, the data processing process is shown in Figure 6.

## 4. Result Analysis and Discussion

### 4.1. Experimental Process of College English Online Teaching Model in Mixed Context

The online practice teaching experiment is carried out based on the actual teaching process of college English curriculum content, and the process analysis is carried out according to the students' classroom performance process. We will set multiple threshold reference values in the neural network algorithm optimized by the Gauss mutation genetic algorithm, so as to ensure that each experimental group can obviously eliminate its inherent data correlation and random error.

**Figure 7:**
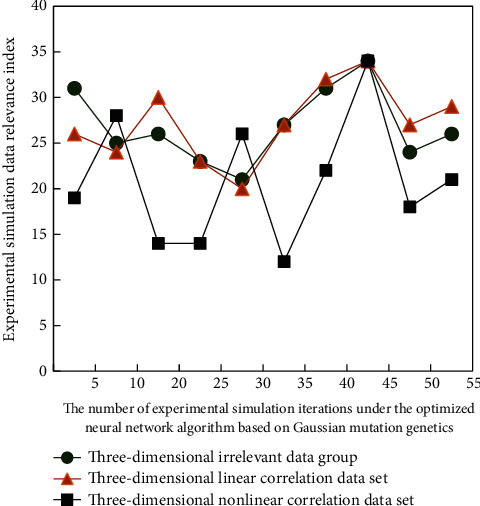
Experimental simulation analysis results.

**Figure 8:**
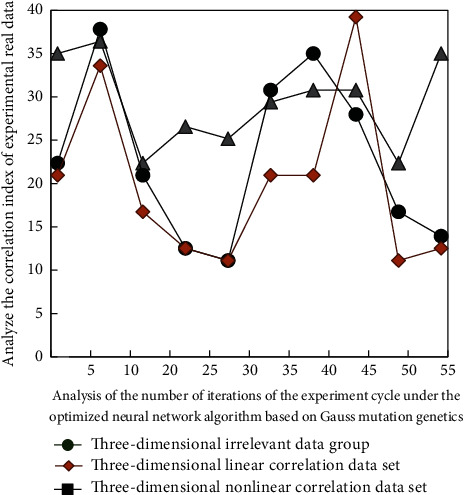
Preliminary experimental analysis results.

On the other hand, the intelligent Gauss mutation genetic algorithm is combined with the optimization of neural network algorithm to optimize its parameters and multivariate analysis. Therefore, in the aspect of data processing, the inherent rules need to be changed. Some improvements are made; the improvement process in the specific experiment process is as follows: the best solution is obtained. The simulation results are shown in Figure 7, and the test results are shown in Figure 8.

It can be seen from Figures 7 and 8 that when the experimental data are determined for different types of data, the internal correlation of the simulation process and the experimental process has a great change, and the change law is also different. This is because the comprehensive solution and correlation analysis of different simulation data groups are not the same, The internal differences can be clearly distinguished, so the change rules are different, which also shows that the online English teaching mode can carry out diversified targeted teaching for students with different English foundation.

### 4.2. Experimental Results and Analysis

**Figure 9:**
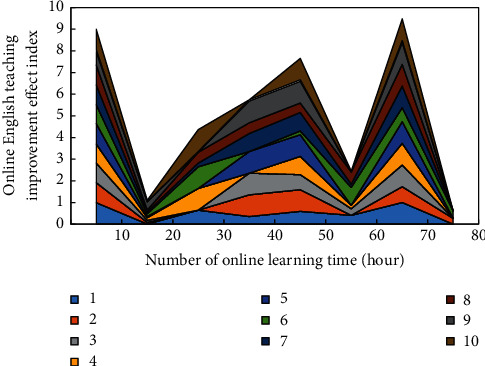
The English improvement effect of different groups in different learning time.

The method of this questionnaire survey is offline one-to-one survey and paper random survey. The survey objects are most professional college students in a university. For college students of different genders and grades, different ways of questionnaire were used in this experiment. The questionnaire includes the following contents: oral English ability, English writing ability, English reading ability, students' classroom participation, homework completion, average grade in class, and so on. During the whole experiment, 91.3% of the participants were satisfied with English classroom teaching, of which 32.2% were girls and 59.1% were boys. In these English tests, 89.3% made significant progress, of which 61% were girls and 39% were boys. The experimental results are shown in Figure 9 (1–10 represent ten groups of students using English online teaching, including 1357 freshmen and sophomores and 2468 seniors, and 9 and 10 groups are boys and girls, respectively).

Through Figure 9, after the online teaching, the students of different grades have a good improvement in their English scores, but the improvement effect of different grades in English achievement is different. This is because the students of different grades have different English foundation, and in learning English courses with different difficulties, everyone's learning ability and learning methods are also different. However, each grade of students can improve the performance of English courses by online English teaching.

## 5. Conclusion

This paper studies the online college English teaching model in the mixed context based on the Gaussian mutation genetic algorithm and neural network optimization. Firstly, it briefly summarizes the current situation of college English teaching quality evaluation, the application of the Gaussian algorithm, and the optimization content of neural network. Secondly, according to the five factors affecting the evaluation of college English classroom teaching quality, it improves the traditional network teaching method. The evaluation method proposed in this paper is more scientific. Finally, through practical tests, the reliability of the evaluation model of network teaching and English classroom teaching quality is quantitatively evaluated. The results show that the method proposed in this paper can not only effectively evaluate the effect of network teaching but also analyze and evaluate the current situation of college students' English ability. However, this paper only focuses on the construction of the college English teaching evaluation model and data category analysis and does not consider how to strengthen the key system of curriculum evaluation. Therefore, before colleges and universities adopt this network teaching method, we can make an in-depth study on the key variable weight evaluation system of English curriculum.

## Data Availability

The data used to support the findings of this study are available from the corresponding author upon request.
